# Supplementary figures and images for: Computational modelling of cell motility modes emerging from cell-matrix adhesion dynamics
Source: PLoS Comput Biol. 2022 Feb 14;18(2):e1009156. doi: 10.1371/journal.pcbi.1009156 (PMC8880896; doi:10.1371/journal.pcbi.1009156)

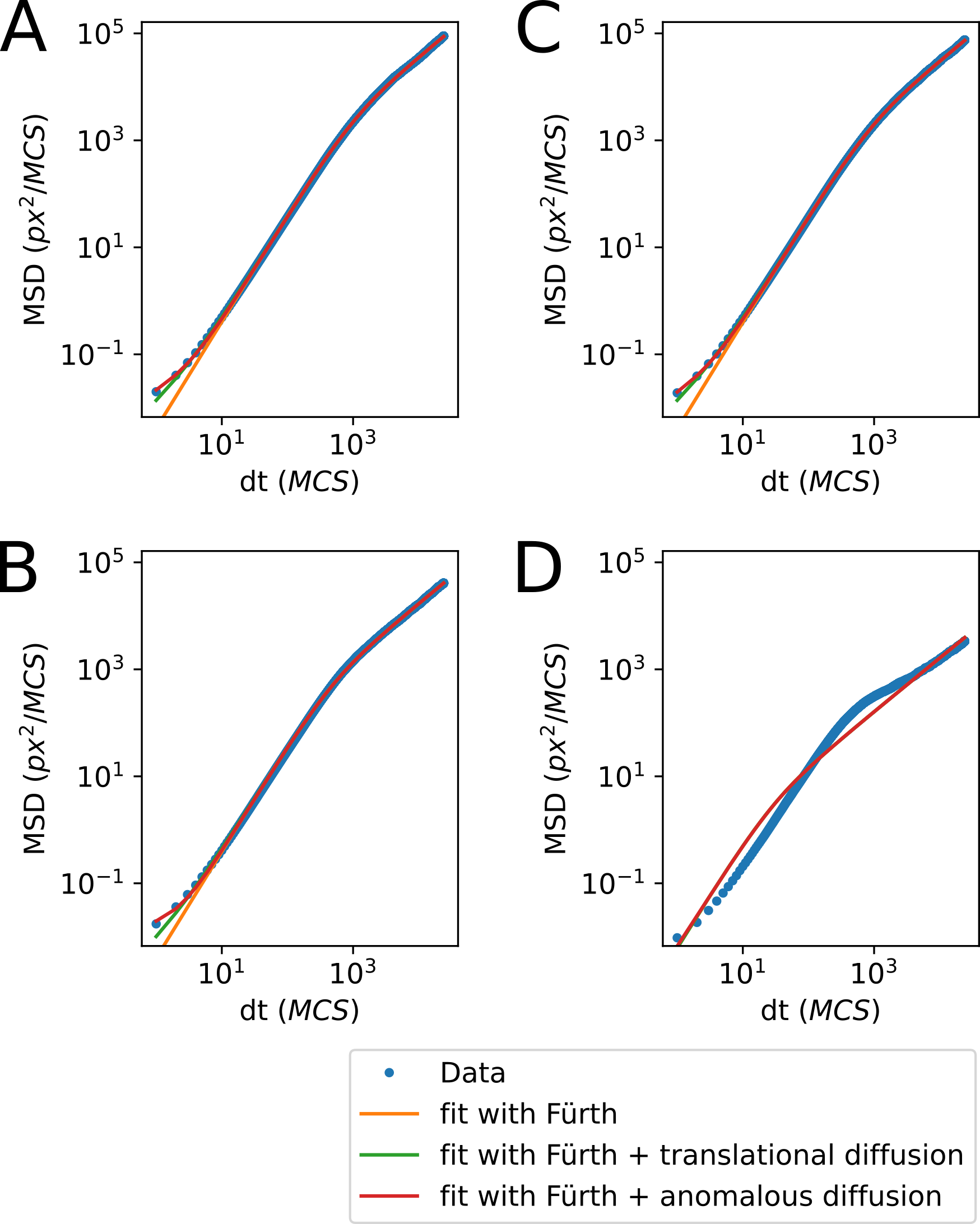

Supplement: S1 Fig — Log-log plot of MSD for the four scenarios in Fig 2, similar to Fig 4, with fits of Eqs 1 and 2. Parameters are: A) λadh = 20, ps = 0.004, B) λadh = 100, ps = 0.004, C) λadh = 20, ps = 0.02, D) λadh = 100, ps = 0.02. (PNG) [file pcbi.1009156.s001.png]

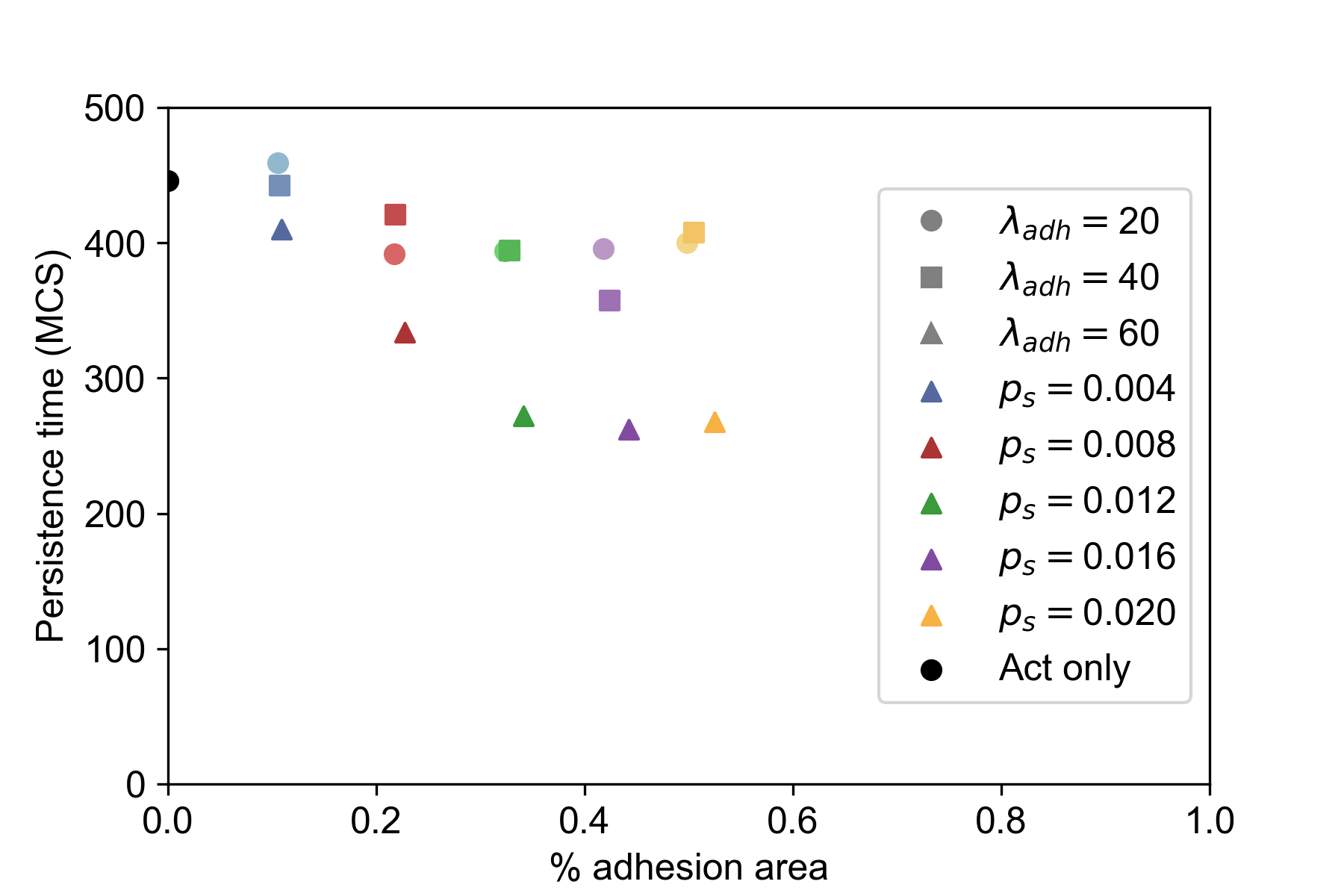

Supplement: S2 Fig — Parameters are the same as in Fig 3, except that λadh has been limited to 20, 40, 60 because of bad fitting with Eq 2. For reference, the persistence time of the Act model without the adhesion extension is plotted as the black dot. (PNG) [file pcbi.1009156.s002.png]

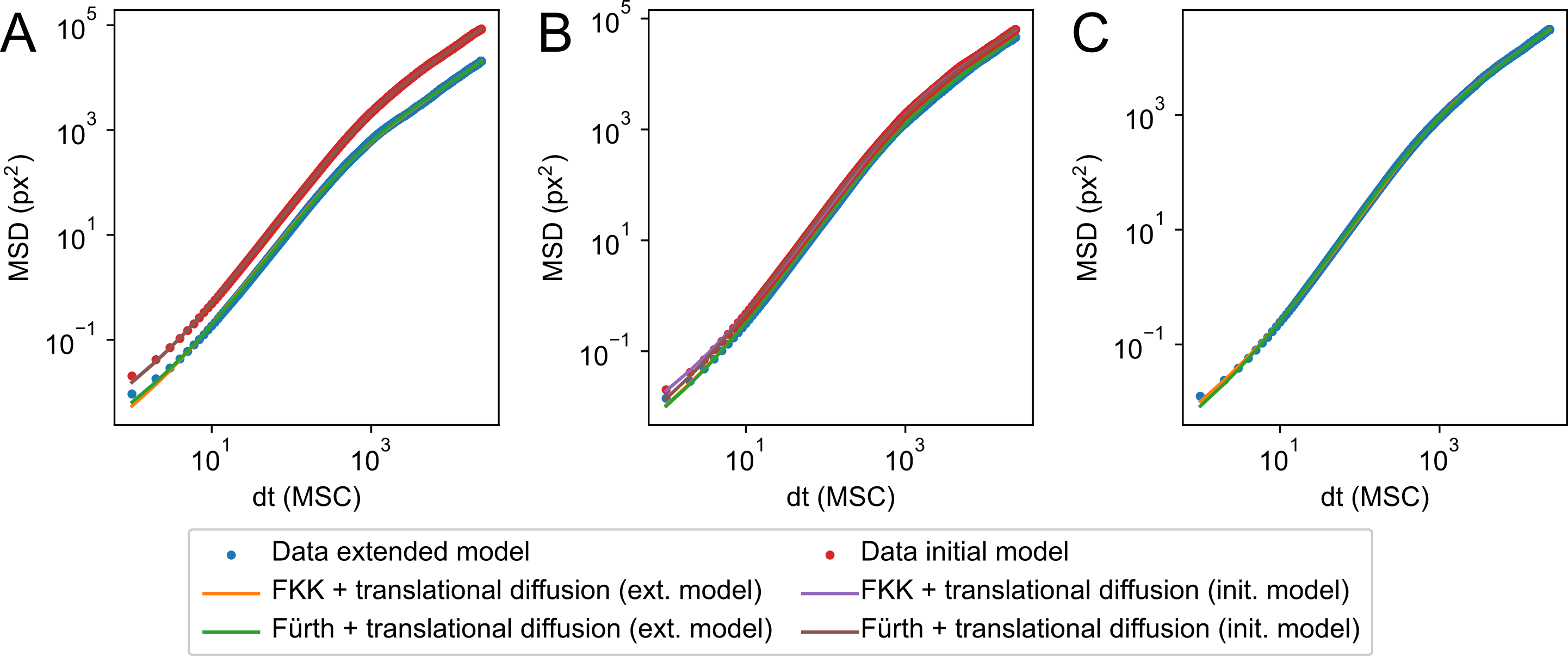

Supplement: S3 Fig — Parameters are the same as in Fig 6, with ps being varied: (A) ps = 0.001, (B) ps = 0.004, and (C) ps = 0.0025. (PNG) [file pcbi.1009156.s003.png]
