# Supplementary material for: Computational modelling of cell motility modes emerging from cell-matrix adhesion dynamics
Source: PLoS Comput Biol. 2022 Feb 14;18(2):e1009156. doi: 10.1371/journal.pcbi.1009156 (PMC8880896; doi:10.1371/journal.pcbi.1009156)
Supplement: S1 Text — (PDF) [file pcbi.1009156.s008.pdf]

S1 Text: Parameter estimations

Computational modelling of cell motility modes emerging from cell-matrix adhesion dynamics

Leonie van Steijn Inge M.N. Wortel Clément Sire
Loïc Dupré Guy Theraulaz Roeland M.H. Merks

In this Supplementary Text, we elaborate on parameter estimation. We first determine the length scale and time scale. After that, we address the parameters involving the Act model and the energy of matrix adhesion.

1 Length scale

The length scale of the model can be easily determined from the area of the cell. We choose a target area of 1000 lattice sites. When we set this area to correspond with the area of B cells and T cells on a substrate which cover an area of approximately 360 μm^2[1, 2], a single lattice site is approximately 600 nm wide.

2 Time scale

With the length scale set, we can derive the time scale from the cell speeds. B cell speeds were on average 8 µm/min in intervals of 5s, or, when measured over 5 minute intervals, 1 and 2.5 µm/min on collagen or fibronectin respectively [1]. This shows that the time interval at which one measures speed greatly matters, and for setting the time scale this means we have a bootstrapping problem of sorts. We decided to measure speeds at 50 MCS intervals, and then our simulated cell speeds vary between 0.02 px/MCS and 0.05 px/MCS. Using the B cell speeds from the 5s intervals, we obtain that 1 MCS=0.1 to 0.25 s, for which the 50 MCS time interval corresponds quite well with the 5 s experimental time interval. Using the B cell speeds from the 5 minute intervals, we obtain 1 MCS=0.3 to 2 s, but then the 50 MCS time interval does not correspond well with the 5 minute experimental time interval. From this, we conclude that a single MCS is in the order of 0.1 s.

3 Comparing Max_{Act} with biological values

The Act model is gouverned by two parameters: Max_{Act} and λ_{Act}. The parameter Max_{Act} is the number of MCS before a lattice site's Act value becomes 0, and can be interpreted as the actin lifetime. We set $\text{Max}_{Act} = 120\text{MCS}$, which corresponds to approximately 20s. Measurements on actin filament lifetimes show lifetimes between 4s and 148s [3] or an average of 27s [4]. Overall, our chosen Max_{Act} corresponds well with these lifetimes.

4 Comparing the actin polymerization and matrix-adhesion energies

The second parameter of the Act model, λ_{Act}, determines the weight of the Act model compared to other terms in the Hamiltonian, and can be interpreted as the maximal protrusive force of the actin network. As

$$\Delta\mathcal{H}_{Act}(\vec{x} \to \vec{y}) = \frac{\lambda_{Act}}{\text{Max}_{Act}} \left(\left(\prod_{\vec{u} \in V(\vec{x})} Act(\vec{u}) \right)^{1/|V(\vec{x})|} - \left(\prod_{\vec{u} \in V(\vec{y})} Act(\vec{u}) \right)^{1/|V(\vec{y})|} \right),$$

and the geometric means are between 0 and Max_{Act}, $\Delta\mathcal{H}_{Act}$ is within the range $[-\lambda_{Act}, \lambda_{Act}]$. Therefore, we can relate the Act work of the protrusion of a single lattice site to λ_{Act}.

Since $\Delta\mathcal{H}_{\text{total}}$ is scaled by the temperature parameter T, the λs are all relative and we cannot attach a biological value to the parameter λ_{Act} directly. However, we can still calculate biologically relevant values for $\Delta\mathcal{H}_{Act}$, the work associated with the protrusion of a single lattice, and compare that with biologically relevant values of the adhesion rupture associated work $\Delta\mathcal{H}_{adh}$. First, we calculate the protrusion work based on values taken from previous literature. From Footer et al. [5], a bunch of eight actin filaments can push with a force of 1 pN. Estimating that a single actin filament is 6 nm wide, and combined with the estimate of 600 nm CPM lattice sites, we conclude that a lattice site (3D) should contain from 1000 to 10000 filaments. Extending the cell with 1 lattice site then begets work of $7.5 \cdot 10^{-17}$ Nm to $6 \cdot 10^{-16}$. A similar estimate can be reached from the measurements of Prass et al. [6]. They measured a force of around 1 nN from the protruding lamellipodium of a keratocyte. This corresponds to a work of $6 \cdot 10^{-16}$ Nm as well.

The work for breaking the bonds of a retracting lattice site, $\Delta\mathcal{H}_{adh}$, is determined by λ_{adh}. In our simulation we use $\lambda_{adh} < \lambda_{Act}$.

We estimate this energy from the measured adhesion forces mentioned in the literature. Gallant et al. [7] measured the force of an adhesion patch of fibroblasts to be around 100 to 200 nN. Considering the length of the retracting lattice site that ruptures the bonds, this results in $6 \cdot 10^-14$ Nm of work to induce rupture. Palecek et al. [8] report a detachment force varying between $1 \cdot 10^{-9}$ and $8 \cdot 10^{-8}$ N in CHO cells. This could result in work terms of $6 \cdot 10^{-16}$ Nm

Table A: List of parameters involved in adhesion dynamics and their units

Parameter	Description	Unit
λ_{Act}	Weight of the Act-extension, the maximum protrusive force induced by actin polymerization	-
Max_{Act}	Maximum value of the Act-field, actin lifetime	MCS
-	Act-value threshold above which adhesion formation is possible	-
p_s	New adhesion formation rate	$(\text{px}^2\text{MCS})^{-1}$
p_e	Rate for neighbouring grid site to become adhesion site if not already so.	$(\text{px MCS})^{-1}$
p_d	Rate of unbinding adhesion site dependent on adhesionless neighbouring sites	MCS^{-1}
λ_{adh}	Energy required to rupture adhesion upon retraction of the cell	-
f	Prefactor for the adhesion feedback onto Act model	-
b	Base value of f in absence of adhesions	-
s	Adhesion area fraction saturation threshold above which $f = 1$	-

to $5 \cdot 10^{-14}$ Nm. Even larger detachment forces are measured by Taubenberger et al. [9] in CHO cells, ranging from 10 to 10000 pN (10^{-8} to 10^{-5} N). A different estimate can be made from single integrin measurements in osteoclasts as done by Lehenkari and Horton [10]. A single integrin can hold 127 pN of force. Estimating that a single integrin is the same as a localization point in the T lymphocyte experiments of Houmadi et al. [2], a lattice site will contain 2200 localization points and thus as many indivual integrins, which would correspond to a total work of $2 \cdot 10^{-13}$.

So we establish that $\Delta\mathcal{H}_{Act}$ is in the order of λ_{Act} in the model and in the order of 10^{-16} Nm derived from literature. Further, $\Delta\mathcal{H}_{adh}$ is set by λ_{adh} in the model, and we estimated it between order 10^{-16} Nm to 10^{-12} Nm using literature based values for adhesion force. So, the adhesion energies are at least equal to the action polymerization energy and at most four orders of magnitude larger. However, this does not correspond with the current settings chosen for the parameter values where $\lambda_{Act} > \lambda_{adh}$. Increasing λ_{adh} to match the estimated ratio between polymerization and adhesion energies will most certainly result in cells stuck to the matrix, as the case where $\lambda_{Act} = 2\lambda_{adh}$ already resulted in pivoting cells.

References

[1] Rey-Barroso J, Calovi DS, Combe M, German Y, Moreau M, Canivet A, et al. Switching between individual and collective motility in B lymphocytes is controlled by cell-matrix adhesion and inter-cellular interactions. Sci Rep. 2018;8(1):5800. doi:10.1038/s41598-018-24222-4.

[2] Houmadi R, Guipouy D, Rey-Barroso J, Vasconcelos Z, Cornet J, Manghi M, et al. The Wiskott-Aldrich Syndrome Protein Contributes to the Assembly of the LFA-1 Nanocluster Belt at the Lytic Synapse. Cell Reports. 2018;22(4):979–991. doi:10.1016/j.celrep.2017.12.088.

[3] Watanabe N, Mitchison TJ. Single-molecule speckle analysis of actin filament turnover in lamellipodia. Science. 2002;295(5557):1083–1086. doi:10.1126/science.1067470.

[4] Smith MB, Kiuchi T, Watanabe N, Vavylonis D. Distributed actin turnover in the lamellipodium and FRAP kinetics. Biophys J. 2013;104(1):247–257. doi:10.1016/j.bpj.2012.11.3819.

[5] Footer MJ, Kerssemakers JWJ, Theriot JA, Dogterom M. Direct measurement of force generation by actin filament polymerization using an optical trap. P Natl Acad Sci USA. 2007;104(7):2181–2186. doi:10.1073/pnas.0607052104.

[6] Prass M, Jacobson K, Mogilner A, Radmacher M. Direct measurement of the lamellipodial protrusive force in a migrating cell. J Cell Biol. 2006;174(6):767–772. doi:10.1083/jcb.200601159.

[7] Gallant ND, Michael KE, García AJ. Cell adhesion strengthening: Contributions of adhesive area, integrin binding, and focal adhesion assembly. Mol Biol Cell. 2005;16(9):4329–4340. doi:10.1091/mbc.E05-02-0170.

[8] Palecek SP, Loftust JC, Ginsberg MH, Lauffenburger DA, Horwitz AF. Integrin-ligand binding properties govern cell migration speed through cell-substratum adhesiveness. Nature. 1997;385(6616):537–540. doi:10.1038/385537a0.

[9] Taubenberger A, Cisneros DA, Friedrichs J, Puech PH, Muller DJ, Franz CM. Revealing Early Steps of $\alpha_2\beta_1$ Integrin-mediated Adhesion to Collagen Type I by Using Single-Cell Force Spectroscopy. Mol Biol Cell. 2007;18(5):1634–1644. doi:10.1091/mbc.e06-09-0777.

[10] Lehenkari PP, Horton MA. Single integrin molecule adhesion forces in intact cells measured by atomic force microscopy. Biochem Biophys. 1999;259(3):645–650. doi:10.1006/bbrc.1999.0827.
