# Supplementary material for: Computational modelling of cell motility modes emerging from cell-matrix adhesion dynamics
Source: PLoS Comput Biol. 2022 Feb 14;18(2):e1009156. doi: 10.1371/journal.pcbi.1009156 (PMC8880896; doi:10.1371/journal.pcbi.1009156)
Supplement: S2 Data — To use the model, open https://ingewortel.github.io/2021-motility-from-adhesion/. Alternatively, download the Supporting Data File (also deposited at https://dx.doi.org/10.5281/zenodo.5914705), unzip the folder “Artistoo” and open Artistoo/active-adhesion/index.html within a web browser. (TGZ) [file pcbi.1009156.s010.tgz › Artistoo/active-adhesion/index.html]

Migration with ECM adhesion


# Interactive simulation: Cell motility through actin-adhesion dynamics

Inge Wortel

The following interactive simulation implements the model from:

Steijn et al (2021). **Computational modelling of cell motility modes emerging from
cell-matrix adhesion dynamics**. *bioRxiv*, doi: 10.1101/2021.06.09.447692

## About the model

See the preprint for model details, but briefly:

- When a cell successfully protrudes and gains a new pixel, that pixel gains an
  **activity** for some time that makes it more likely that this pixel will protrude
  again (**positive feedback**, activity indicated by red-yellow color below).
  The duration of the "activity memory" is governed by
  maxact, the strength of the positive feedback by λact;
- In the protrusive region where local activities are >0.75 x maxact,
  **adhesive sites** can spawn with probability ps. Such adhesive sites
  (dark pixels in the simulation below) are
  harder for the cell to detach if it wants to retract, so copy attempts into an
  adhesion get penalty λadh;
- Adhesive patches can also **expand and decay** independently, at rates governed
  by the parameters pe and pd, respectively;
- Adhesion to the surface determines how well the cell can translate protrusive
  force at the front into actual motion, because **higher adhesion prevents "slipping"**
  and staying in place. The fraction of the cell surface occupied by adhesive sites
  therefore modifies λact: the effective λact
  starts from a baseline fraction *b* of its value when there are no adhesions
  at all, and reaches its full value only when at least a fraction *s* of the
  cell surface is adhered to the substrate.

## Try it yourself

You can use the parameter sliders in the dropdown menu below ("simulation parameters")
to explore how changing them affects cell motility. Yellow to red colors indicate
active pixels with protrusive feedback. Dark patches are adhesions, and the blue line
shows the trajectory of the cell's center of mass over time.

Simulation parameters

Act-model:

λact

Scales the strength of the protrusive feedback in the Act-model.

maxact

"Memory" of the protrusive feedback.

ECM interaction:

λadh

Penalty for copy attempts into a stable adhesion.

ps (x10000)

Probability of spawning a new adhesion in regions with local activity > 0.75 x maxact.

pe (x10000)

Probability of adhesion expanding into empty neighbor site during Eden growth.

pd (x10000)

Probability of deleting an existing adhesion, per adhesion-free neighbor site.

Feedback on protrusive strength:

b (x1000)

Baseline multiplier for λact.

s (x1000)

Fractional adhesive area at which (the multiplier for) λact saturates.

  

Simulation controls  
The random seed only takes effect when you reset (  ).
The same parameters + random seed should yield the exact same output every time.
If you tick "record track", then the cell's center of mass will be tracked
in the 'outputs' menu below.

Seed: 
  
   
Record track
   Visualize track: draw every
 MCS, remember
 MCS.

Simulation powered by Artistoo.

Outputs
(Data from the simulation, only when 'record track' is ticked above)

Cell tracks:

Suggestions to try:

- When either λact or maxact is zero, cells should
  stop moving because there is no "protrusive feedback" or "activity memory".
- When λadh is very high, cells should stop moving because
  their adhesions are anchored so firmly that they are impossible to detach.
- The number of adhesion patches can be controlled by
  ps, pe, and pd. Higher ps/pe
  should lead to more adhesive patches because of higher formation rates, and lowering
  pd has the same effect by making existing patches more stable.
- ps and pe also control the spatial distribution of adhesions:
  when only ps > 0, patches are smaller and more evenly distributed because
  existing patches cannot expand. By contrast, when ps is lower and pe
  is very high, patches tend to be larger and can accumulate at the rear of the cell,
  slowing it down and/or making it "pivot" and turn.
- when b = 1 and s = 0 (the default), the cell does not slip on the surface
  regardless the number of adhesions. Setting b = 0 and s = 1 should make the
  cell dependent on adhesions for motion: at these settings, setting ps/pe
  to zero and pd to high values will stop motion.

## See also

The preprint:

Steijn et al (2021). Computational modelling of cell motility modes emerging from
cell-matrix adhesion dynamics. *bioRxiv*.

About the Act-model this model was based on:

Niculescu et al (2015). Crawling
and Gliding: A Computational Model for Shape-Driven Cell Migration. *PLoS Computational Biology*.

Wortel et al (2021). Local
Actin Dynamics Couple Speed and Persistence in a Cellular Potts Model of Cell Migration. *Biophysical Journal*.

More interactive explorables:

Explorable: Act-model

Explorable: Introduction to the CPM
