# Supplementary material for: Computational modelling of cell motility modes emerging from cell-matrix adhesion dynamics
Source: PLoS Comput Biol. 2022 Feb 14;18(2):e1009156. doi: 10.1371/journal.pcbi.1009156 (PMC8880896; doi:10.1371/journal.pcbi.1009156)
Supplement: Attachment — Submitted filename: Response-to-reviews.pdf [file pcbi.1009156.s012.pdf]

1 Response to Referees

Code: PCOMPBIOL-D-21-01092
Title: Computational modelling of cell motility modes emerging from cell-matrix adhesion dynamics
Authors: L. van Steijn, C. Sire, L. Dupre, G. Theraulaz, R.M.H. Merks

1.1 Reviewer 1

This paper uses a cellular Potts model (CPM) to explore how cell adhesion to surrounding extracellular matrix (ECM) affects the migratory behavior of the cell. The modeling and computing methodology appear to be sound, the exposition of the results is largely clear, and the research seems to have been executed with care.

Thank you for the nice remarks

However, a flaw of the presentation is that it does not clearly state the unique contributions of this model vis-à-vis previous modeling work. The Introduction motivates the work by an open question: what factors cause the different modes of motility? It then argues that these should include both the intracellular actin dynamics and the cell-ECM adhesion. But in the end (L. 92-94), "we are able to reproduce a variety of cell motion types, similar to the behaviour seen in other models that also include those two components [15-17]." This does not represent a novel and significant contribution toward answering the open question.

Thank you for pointing out this inconsistency in the description of our aims. We have now clarified our aims vis à vis the previous work through a full rewrite of the introduction. Briefly, the aim is to understand how bulk cell movement, e.g. subdiffusive and diffusive motility, and hence immune cell search strategies, follow from a balancing act between the cell-instrinsic mechanisms of cell motility and the kinetics of cell-ECM adhesion. Previous experimental work and theoretical modeling has already given much insight into this, but computational and experimental limitations prohibited measurement of bulk motility characteristics as a function of cell motility and cell-ECM adhesion. Thanks to our new, highly efficient model this is now possible demonstrated in the in-depth analyses followed by characterization of the MSD curves and velocity distributions

The Results (pp. 9-20) are clearly presented but contain few surprises. In particular, there is no discussion in this section of how this new model advances our understanding beyond what was known before.

The key surprise and novelty of the work is that with a highly coarse-grained, yet fully mechanistic model we can reproduce salient features of non-diffusive cell motility, as discussed above

Finally, the Discussion section is long and discursive. Besides a summary of the key results, it touches on many things including parameter variability, the time scales, and potential improvements on the current model. But it does not address the issue of novelty and significance of the current work against the backdrop of earlier modeling.

We have rewritten the first paragraph of the discussion, but requests by the other reviewers made it necessary to make some further additions as well.

More detailed questions:

1. Abstract: "With an increasing attachment area and increased adhesion strength, the cells' speed and persistence decreases." This sounds reasonable, and is later born out by Figure 2. But this seems inconsistent with the experimental evidence that B lymphocytes form "larger adhesive connections" on fibronectin than on collagen, but move faster on the former (L. 22-25).

   This point is revisited in Discussion (L. 416 onward), but I was unable to understand the explanation or rationalization that follows. Is this a failing of the model? In which aspects? What may be the factors that produced the experimental observations that are unaccounted for?

   This is indeed a failing of the model. We've now stated this explicitly with the sentence. The section has been rewritten as follows: "Despite these agreements with experimental data, clear disagreement of the model predictions with experimental observation of course remains. B cells form larger adhesive connections on fibronectin than on collagen IV, and they move faster and more randomly on fibronectin, whereas they move more slowly and more persistently on collagen IV [1]. Interestingly, this observation also disagrees with the universal coupling between cell speed and cell persistence (UCSP) [2] which is reproduced by the Act-model [3] that is at the basis of our model. Briefly, UCSP states that persistence correlates positively with speed. Future experimental and theoretical work will hopefully elucidate the causes of this counterexample to the UCSP principle; possibly causes might include polarization cues in the collagen IV matrix facilitating persistent motion, or activation of actin polymerization in response to matrix binding: integrin type $\alpha_5\beta_1$ signalling induces membrane protrusions, and generates RhoA/Rock-mediated myosin II, whereas α_v integrins signal to reinforce adhesive sites [4]."

2. L. 52-54: does the model explain the apparent contrast between D. Discoideum and T cells cited here? I didn't see where the paper returns to this discrepancy later.

   Thank you for pointing out this issue. The reviewer is right, our model does not help to resolve this discrepancy, and we realize the statement

distracts readers from the flow of the argument at this point. We have deleted the statement here in the introductions.

3. L. 88: "In a second version of the model". At this point, I wonder why 2 versions of the model are presented. Much later on p. 16 (L. 257 onward), we learn that the extended model captures all 3 modes of migration (i - iii on L. 249-251). Then why include the lesser version of the model at all?

   We prefer to present the work in this manner, to show how simple assumptions already reproduce much of the complexity of anomalous diffusion of immune cells. By introducing a new assumption only when we need it, we make clear how each aspect contributes to the dynamics of the model. We have clarified this approach at the end of the introduction

4. L. 108: The subsection on "Modelling cell-matrix adhesions" does not present results and should be in the Methods rather than the Results section. *In fact this a commonly used approach in papers in which the Methods section appears in the end, and we prefer to follow it. This section introduces the key assumptions of the new model in general terms such that the rest of Results section can be followed without referring to the Methods section. The Methods sections provides all details necessary for implementation. The alternative is to switch to the traditional ordering Introduction, Methods, Results, Discussion, which would then make it possible to move this section to Methods without interrupting the flow of the story. However, we strongly prefer the present structure in order to separate the key points and the details.*

5. L. 127: I found the idea of "single adhesion units" intriguing. Is this a single molecular bond? Perhaps this can be explained quickly so the reader need not consult [23].

   No, it does not contain a single molecular bond. The idea of 'single adhesion unit' turned out to be ill-defined, and the reasoning turned out to be based on an unrelated type of integrin. Thank you for catching this issue. We have revised the text accordingly, as follows: "The integrin bonds through which the cell adheres to the substrate are represented by a binary number in each lattice site, which indicates if it contains active adhesions or not. "

6. Caption to Fig. 2: First, the caption should identify the "different motility types": what they are, and to which row they correspond. Second, the 5 vertical dash lines mark the times for the snapshots in the first column. Which corresponds to the snapshots in the second column?
   We extended the caption to include descriptions of the observed behaviour seen in A-D, and a line stating which times correspond to the snapshots in the second column.

1.2 Reviewer 2

In this paper, the authors incorporate the mechanism of cell protrusion and cell-matrix adhesion into Cellular Potts model and study the relationship between different cell moving modes with those microscopic process including adhesion formation, growth/shrinkage, feedback of adhesion to protrusion, etc. Basically, the questions this paper is focusing on are interesting and Cellular Potts model, as mentioned by the author, is a proper model to study these questions because of the light computational cost and the incorporation of different forces related to cell's movement. But there is one major concern about using 2D Cellular Potts model to study questions related to adhesion and several comments on the way they show their results, which makes the paper less informative and convincing.

1. The main concern about the 2D Cellular Potts Model: one main characteristic of CPM is that the dynamics only happen at the boundary of the cell (where $\sigma(x)$ is not the same as $\sigma(y)$), which is also mentioned in the Methods part of this paper. This means only the adhesion sites at the boundary play a role in the movement of the cell. So, I think it is not proper to relate the cell's movement to the overall adhesion area and adhesion distribution as most of the adhesion sites taken into account in fact do not function/do not enter the ΔH at all. In my opinion, it is better to use a 3D model when the topics are related to the attachment/detachment of the cell to the substrate as then there is a real "surface" that contributes to the adhesion. While now in 2D, it is hard to say whether the interior of the cell contacts with the matrix or not: they cannot exchange sites with the outside but they have adhesion sites. I understand that 3D simulation will greatly increase the computational cost and I do not totally contradict to the use of 2D model but the interpretation should be more careful. The authors need to give reasonable explanations on the interpretation of adhesion with matrix in 2D model and think about how to calculate the "percentage of adhesion area" again. If this explanation is not well given, the fundamental of the model is doubtful.

   We acknowledge this concern about the interpretation of this two-dimensional model of a three-dimensional problem, and hope to have clarified this issue in the second paragraph of the section Results:

   "In this two-dimensional model we consider integrin bonds between the cell and the underlying substrate, by which the cell adheres to the substrate. They are represented by a binary number in each lattice site, which indicates if at this site there is an active adhesion or not. In biological cells, the formation of new adhesions occurs at the cells leading edge during actin polymerisation and pseudopod protrusion [5, 6, 7], as well as through formation and expansion of adhesions in the middle region of the cell. In absence of forces pulling the whole cell off of the substrate, due

to the two-dimensional representation of the cell, only the adhesions at the edges of the cell will affect cell motility. Nevertheless, we will need to consider formation and degradation of adhesion in the middle of the cell as well, because–as will become apparent when we consider the dynamics of the model–a large patch of adhesions at the cell edge will still resist as series of unbinding events better than a few isolated adhesions. In addition, in an extended version of the model (see Section ...) we included an effect of the adhesions on actin polymerization."

Also we have added a clarification of 'adhesion area percentage' as follows:
"... regulates the adhesion area, as characterised by the 'adhesion area percentage', i.e. the percentage of the projected cell area that contain adhesions to the substrate (Fig. **??***). "*

2. The authors mentions several moving modes of the cells and the three that they are able to obtain with their model are the floating, sliding-stepping and pivoting. The differences they show to discriminate these three modes are the opposite dependence of speed on adhesion area and the sub-diffusive behavior shown in MSD. To my knowledge, these are not the only difference between these modes. For example, there are possibly morphological difference and in stepping mode, there is dynamic attachment/detachment behavior. Of course, it is not fair to require one model to capture all the characteristics but it is necessary for the author to write more clearly what are the main differences of these three modes and which of them can or cannot be captured by the current model. Without this, even I believe the speed and MSD data they show, I do not know to what extent I can relate these three "modes" in the simulation to the three "modes" observed in the experiments. After all, it is possible that not one moving mechanisms show similar speed-adhesion are relation or similar MSD.

   The reviewer asks to describe more clearly the differences between the cell motility patterns predicted in the model and the modes observed in experiments. We have added explanation of the role of the cell-substrate adhesions at the end of section "Model reproduces walking motility and sustained attachment leading to pivoting motility":

   An important difference is that the gliding cells in experiments show a large adhesion patch at the front where they assist in cell protrusion, whereas in our model the bulk of the adhesions appears more to the back, where they mostly reduce cell retraction probability (Fig **??***(C,G)).*

   and in the beginning of section "Including the effects of cell-substrate adhesion on cell protrusion":
   However, key differences between the behavior of the extended Act-model

with real cell motility remain. In particular, in the extended model the cell-substrate adhesions only reduce the probability of cell retraction, whereas in biological cells, the adhesions near the cell front are instrumental for pulling the cell forward.

3. The right panels of Figure 2 and Figure 6 are not informative enough to support some of their conclusions. It is better to add statistical measurement (e.g. mean and std). For example, in line 181, they mention that the speed fluctuates less in 2E,G than in 2F,H, which is not obvious in the current noisy curves. A way to quantify the fluctuation is needed. And the stick-slip-like behavior mentioned in line 182 and 183 is not obvious, too, considering the fluctuation of instantaneous speed is large and this is only one example among so many simulations.

   *We agree that statistical measurements for Fig 2 E-H and Figure 6 D-F are required. We have added a line and a shaded area in the figures to indicate mean and std of both speed and adhesion area %. We noticed that the cell speeds do not follow a normal distribution as shown by the speed distributions that we hvave added Fig 2. We have now changed the observation of sliding-stepping-like behavior to: "Indeed, at relatively low values of $\lambda_{adh} = 20$ (**??**E,G) the average speed was higher and also fluctuated less than at higher values of $\lambda_{adh} = 100$ (**??**F,H), as indicated by the mean squared logarithmic difference (E:0.00313 and G:0.00442 versus F:0.0072 and H:0.0167; see distributions at the left). λ_{adh} thus negatively correlated with cell speed. Interestingly, in (Fig **??**B,F we observed sliding-stepping-like behavior: the cells frequently slow down followed by burst of acceleration. However, the simulations do not display the cyclic expansive and contractile shape changes observed in experiments [1] and reproduced in more complex models [8]." In order to support the statement of the fluctuations in speed, we have measured the mean squared log differences of the speeds shown in Figure 2.*

4. In the section beginning from line 298, the authors mention that they want to study the effect of different distribution of adhesion sites. In line 299, they say that they have mostly looked the effect of adhesions formed at the cell front but in Fig.2, for example, I see lots of adhesion sites in the center and back of the cell. So this sentence is not clear.

   We clarified this in the text:

   So far, we have neglected the effect of adhesion distribution and variation in time on cell motility. However, the dynamics of the distribution and size of the adhesive patches over the cell may have a large effect on cell motility patterns.

In Fig.8, they only show the distribution of adhesion cluster size but no statistic information about the sites position (whether mostly at the cell front or other parts) but they conclude in line 325 and 326 that "where those cluster are located" also influences cell motility. What they show in Fig. 8 do not support this conclusion directly. Although I believe it is true because p_s is large, it is better to show the location distribution of the adhesion sites.

We have analyzed the distribution of the adhesions along the cell's directional axis. The new data have been include as panel 8C and support the earlier statement.

5. Still Fig.8, the difference, especially in 8D, is so small. I wonder what prevent the author from choosing another set of parameters that differ from each other more and show larger difference. And in the text, they say that this difference is caused by the distribution (position and size) of the cluster rather than the total adhesion area because the parameters they choose result in the same adhesion area (line 307). But based on their Fig.2 and Fig.6, the adhesion area is a noisy and changing value and thus it is impossible to keep them exactly the same. Then in Fig.8, the difference between the orange and blue is so small. How can they rule out the possibility that this small difference is caused by the small difference in adhesion area?

   The reviewer correctly notes that the differences between the curves in 8D (now 8E) is very small. Please note that the plot is on a log-log scale, such that the difference at the onset of the final, linear regime is by a factor 2. Then the reviewer asks how we can rule out the possibility is caused by the small difference in adhesion area. We have added a new panel B showing that there is very little difference in adhesion area, and we have nuanced our conclusion ("Although we cannot fully exclude that...")

1.3 Reviewer 3

The authors study theoretically the impact of cell-matrix interactions on biological cell migration. Their work is motivated by experimental findings reported particularly in their Ref.[12], to some extent also in [13], where different modes of migration were observed for lymphocytes on different substrates.

To qualitatively reproduce these findings, they start from a cellular Potts model as introduced in the widely cited Ref.[21], which more recently was amended by a more detailed modeling of the actin dynamics, called Act model [19,20].

The authors amend the Act model by capturing further microscopic details of the cell-matrix interaction, as nicely pictorially summarised in Fig.1. Their generalised model is solved numerically by generating cell paths on a plane, which are statistically analysed in various ways such as, e.g., by computing the mean square displacement (MSD). The authors argue that their model qualitatively reproduces the three main modes of cell migration experimentally observed in [12,13], and that it may pave the way to understand further details of the cell-matrix interaction when matching it to further experiments.

First of all, I may remark that I am not a cell biologist but a theoretical physicist, who applies methods of nonequilibrium statistical physics and stochastic processes to understand cell migration. Hence I cannot judge more intricate biological details but rather focus on the stochastic modeling aspects of this work. In my view, this paper addresses an important problem in the field of cell migration, which is to resolve further details of the cell-matrix interaction that in turn crucially determines cell migration.

The paper starts with a very good introduction motivating this problem by suitably embedding this research into the respective field. There are a few minor technical issues here that I will comment on below, but altogether the introduction looks fine. The claim on p.6 that 'this model can reproduce a wide range of lymphocyte motility modes' is a rather big one, but the constructed model, including four new microscopic adhesion mechanism, cf. Fig.1, looks rather complex and quite versatile. Concerning the numerical analysis, I am afraid something went wrong in view of detecting the subdiffusive regime, as I will argue in more detail below.

We thank the reviewer for their nice remarks. The claim on p. 6 has been toned down to 'a number of motility modes'

And while the proposed model reproduces three different motility phases on different time scales (two of them being transient), by construction I do not see that it helps to understand the emergence of non-Markovian non-Gaussian long-time superdiffusive cell migration as experimentally observed, e.g., in [4,32] and many other publications by now, see further references in [4,32] and new citations to these papers. The authors discuss this problem, to some extent, in their conclusions on p. 24ff. After all, the model that is studied in this ms. appears to be an essentially 'Markovian' model, if I am not mistaken.

The reviewer is right that at microscopic level the CPM is Markovian. However, note that the dynamics of the model introduces a memory through the Act values and the adhesion sites. This memory makes the behavior of the simulated cell as a whole non-Markovian, as we now discuss in more detail.

In summary, I do think that this paper forms an important contribution to the field of cell migration by pushing previous models of cell-matrix interactions

generating cell migration to the next level. Still there remain quite a number of important open questions, as indicated above, so I would not consider this model to yield the 'ultimate answer'. But in my view this ms. may be publishable in PLoS Comp. Biol. if it is suitably revised, as further explained below.

1. p.3, l5ff: There is a confusion here between 'obstacles' and 'targets'. If I am not mistaken (the authors may correct me if I am wrong), only in [1] the impact of 'obstacles' (viz. topology of the environment) on search has been studied. In [2-5] the environment has only been crowded with 'targets'. The difference is that a searcher will be hindered by an obstacle in some way to pursue its path while a target has no (or 'little') impact on the dynamics, it just needs to be detected.

Thank you. We agree that this part was not clear and we have rewritten it accordingly

Furthermore, a target may be 'destroyed' (food source emptied) or 'replenished' while an obstacle remains as it is. I appreciate that the distinction is not always perfectly clear depending on the details of a model, but in principle these are two different characteristics, and this needs to be clarified. In particular, in almost all cases I know of, only the dependence of search efficiency on the density of targets has been studied, where the latter are, again, typically not 'obstacles'. Seems this discussion has been 'bent' a bit to better motivate the problem of cell-matrix ('obstacle') interaction.

Yes, this introductory paragraph indeed required some more attention. We have revised it following the suggestions of the reviewer.

2. p.3, l10: Subdiffusive search is mentioned here, but no reference is given to any relevant theoretical works (only to experimental results [9]). Indeed, testing efficiency of subdiffusive search appears to be a more recent problem, but there is, e.g., the key Ref.[A] below as well as the very recent Ref.[B] with further references therein.

Thank you for these missing references to theoretical works on the efficiency of subdiffusive search; we have used these to revise the discussion of subdiffusive search strategies starting from l18.

3. p.6, l98: Here the authors refer to [12] in view of the three phases of cell migration they wish to detect. I looked at this reference, without reading through it in detail, but could not find any clear evidence for subdiffusion in it, in terms of a MSD that increases with time slower than linear in the long time limit. Did I miss anything here?

This has been rewritten.

4. p.10, l195: Computing 'diffusivities' presupposes that the MSD increases strictly linearly in time. But whether this holds is not clear at this point. I suppose the time range given in l202 refers to Fig.4, which is only discussed much later. Perhaps this can be streamlined somehow. Particularly in view of the introduction, where long-time superdiffusion was discussed, this looks a bit ambiguous.

Indeed, at the point where we introduce the diffusivity measure, we haven't characterized the statistical motion yet, which we do in the two sections after that. We've changed the sentence on how we obtain the diffusivity to "As a crude measure for diffusivity we took the slope of the mean squared displacement curves from 11500 to 24000 MCS, assuming the MSD has entered a linear regime in that time period as discussed in more detail below."

5. p.13ff: The MSD of the Fürth equation (1) is nothing else than the solution of what physicists call an (underdamped) Langevin equation, which models what physicists call Brownian motion. 'Persistent random walk' is another name for it. As such, Eq.(1) has a short-time ballistic regime $\sim t^2$ with a cross-over to long-time normal diffusion $\sim t$, which yields two different dynamical regimes. The authors add another linear MSD term in Eq.(2) to better fit their simulation data, which indeed counteracts the ballistic phase on short time scales yielding a new third short-time normal diffusive regime. Per se, this model cannot reproduce any non-trivial memory effects viz. long-time anomalous dynamics.

We agree with this explanation so far.

This is just a brief summary of the situation here. Now the authors take Eq.(3) from [32] to model subdiffusion, amended again by a linear time dependence in (4). They find that an exponent of $\alpha > 1$ reproduces the data well. Meaning (4) yields a MSD linear in time for short times, a crossover to intermediate superdiffusion but then long-time subdiffusion, see Fig.4.D.

Agreed

Unfortunately, the FKK model used in Ref.[32] from which (3) has been obtained is only valid for $\alpha < 1$ and not for $\alpha > 1$, as the authors may check [C]. That means the FKK strictly only models superdiffusion but cannot yield subdiffusion, as the fractional derivative therein is not defined in this parameter regime. Eqs.(3,4) may nevertheles be solutiuons of the MSD for some other stochastic model, I suspect an underdamped Langevin equation with (coloured) Mittag-Leffler correlated noise plus some additional short-time normal diffusion, but to further elaborate on this is a different story. In summary, unfortunately (4) cannot be used with reference to [32], respectively [C]. As it stands, this is

a purely phenomenological equation which would need to be justified by some other stochastic model.

We thank the reviewer for pointing out the restriction to $0 < \alpha < 1$ *in [32] and [C]. We've only used Eq. 4 for fitting, not for stating that the underlying stochastic model describes the motion. However, we have also found a different stochastic model by Jeon and Metzler, a fractional Langevin equation, that yields the same expression for the MSD, but holds for* $1 < \alpha < 2$. *We've added the limitations of* α *to the description of Eq. 3 and added the sentence: "The same expression, except for the noise term, has also been derived from a fractional Langevin equation of motion, which only holds for* $1 < \alpha < 2$ *[Jeon&Metzler]"*

Two further related remarks: 1. The linear fit for the shortest times appears to be reasonable, but instead the authors could try to add $D_T t^\beta, 0 < \beta < 1$ to (1), which would model subdiffusion on short time scales with a generalised diffusion coefficient D_T, dominated by Brownian motion for longer times. 2. Based on see above, I am rather skeptical about the claimed evidence for long-time subdiffusion. The bending off of the MSD in Fig.4.D could just indicate a crossover to normal diffusion for longer times, cf. Figs.4.A-C. I appreciate that eventually cells may completely 'get stuck' at some point, but how precisely this transition from diffusion to localisation occurs, i.e., via (transient) subdiffusion, asymptotic long-time subdiffusion, etc., remains rather unclear to me. I would encourage the authors to study this problem a bit further, as explicit claims are made in the current ms. based on the fit with (2) which, as explained above, is not quite substantiated. There is an open problem here.

We have also fitted the MSD with Fürth + $D_T t^\beta, 0 < \beta < 1$ *and included the fits in Figure S1, and added the sentence "Extending the Fürth equation with anomalous diffusion instead of translational diffusion, i.e. adding the term* $D_T t^\beta$ *does not result in better fits, despite the additional parameter (Fig S1)."*

The Fürth equation describes the mean squared displacement of a particle of position x *in the 2D plane, whose velocity* v *follows the Ornstein-Uhlenbeck process. The 4-dimensional process* $(x, v)(t)$ *is indeed Gaussian and Markovian in 4 dimensions... but the 2-dimensional (projected) process* $x(t)$ *is Gaussian but not Markovian and is not equivalent to the Brownian motion (the only Gaussian and Markovian process, up to a linear transformation; see also our answer to point 6). The fact that the velocity has a finite memory (true, the temporal correlation function is a simple exponential whose decay is characterized by only one temporal scale) results in the non-Markovianity of the position. This process is indeed the simplest Gaussian and non-Markovian persistent process (correlated velocity, and hence displacement) in opposition to the Brownian motion (uncorrelated displacement, and hence Markovian).*

6. p.24: This is an important discussion. As mentioned at the beginning of my report, in my view there is ample evidence for superdiffusive cell migration

on 'asymptotic' very long time scales (amply explained in this paper), which per se cannot be reproduced by the model that the authors put forward here. (Though there is a 'philosophical' discussion of whether eventually all anomalous dynamics may die out by becoming 'normal', which would imply that all anomalous dynamics must be transient.) Mathematically, I would say that their model is essentially 'Markovian', though there may be 'collective adhesion effects' that may be more non-trivial. But probably these all die out on long time scales, which would explain why eventually all these cells seem to exhibit normal diffusion. Again, the transition to localisation is more subtle, and whether there is any non-trivial long-time subdiffusion is, as it stands, unclear, at least to me. But if the dynamics that is generated here is Markovian and Gaussian, there is no way to reproduce any non-trivial non-Markovian and non-Gaussian superdiffusion. So here this model may get to its limits, which perhaps should be more clearly pointed out.

Note that although the microscopic rules of the CPM are Markovian, the Act-levels and growth of cell-ECM adhesions feed-back on cell motility, and hence provide introduce a correlation that introduces non-Markovian behavior.

The fact that the individual Potts variables dynamics is generated from an intrinsically Markovian Monte-Carlo method ***does not at all imply*** *that the overall dynamics, for instance for the center of mass of the cell, is Markovian. Indeed, a local change in the cell boundary at some time can later influence another change elsewhere on the boundary.*

To illustrate this, let us take an even simpler example than our system. Let us start from a random distribution of a large number of particles on a one-dimensional lattice (with periodic boundary conditions), and at each Monte Carlo step, let us select a particle at random and move it one lattice site to the right or left with probability 1/2, an intrinsically Markovian dynamics. It is easy to show that the density of particles (minus the average density) occupying a given site of the lattice, seen as a temporal random process, is not a Markovian process (despite the density field being described by the simple diffusion equation; this very interesting non Markovian process is in particular studied in Phys. Rev. Lett. 77, 2867 (1996) (https://journals.aps.org/prl/abstract/10.1103/PhysRevLett.77.2867 involving one of the present author).

Another example related to point 5 of the referee is a particle of position x moving on a 1D line whose velocity v follows the Ornstein-Uhlenbeck process. The two-dimensional process (x,v)(t) is indeed Markovian in 2 dimensions... but the one-dimensional (projected) process x(t) is not Markovian. For instance, because of its non-Markovianity, the calculation of the probability for this process to remain positive up to time t is a very hard mathematical problem solved by Burkhardt in 1993, whereas it is a trivial problem for the Brownian (and hence Markovian) process (see the reference [16] in the reference provided above).

Physical observables (like the position of the center of mass of our cells) projected from a (very) high-dimensional Markovian dynamics have simply no reason to have a Markovian dynamics or even short-time memory. This is "fortunate", since once the Hamiltonian of a real system is known, Monte-Carlo dynamics faithfully describe its statistical dynamics... including for physical systems displaying long term memory (think of spin glasses as an extreme example of "infinite memory" systems despite being described by a Monte Carlo dynamics).

Indeed, as is also discussed on p.25ff, biologically a reason may be that non-trivial cytoskeletal fluctuations are not properly taken into account in this model. As far as I remember there are experimental results (unfortunately I don't have a good reference ad hoc) indicating that the cell cytoskeleton is a complicated biopolymer gel with anomalous (power-law) rheological properties. While I appreciate that the authors' model captures a lot of relevant details of the cell-matrix adhesion process, I think it is rather generous as far as this second important part of the cell migration process is concerned, as to some extent the authors discuss on p.25. Meaning I do not necessarily see any contradiction here, but perhaps the range of validity and application of the present model needs to be discussed a bit more thoroughly in view of reproducing observed long-time anomalous diffusion and details of the cytoskeleton dynamics possibly driving these anomalies. One way forward might be a non-Markovian stochastic input into the model at a suitable point, which may inject non-trivial memory in the dynamics.

We agree that the model as is cannot account for the long-term superdiffusion, possible because we lack a system for even longer-term correlations. We added to the last sentence of the discussion on long-term superdiffusion: "although non-trivial behaviour among the factors that determine cell polarity such as the cytoskeleton can play a role [Abaurrea Velasco et al., 2017], and have not been addressed by this model". The article by Abaurrea Velasco et al. shows a minimal model of cytoskeletal driven cell motion and is able to display long-term superdiffusive motion.

[A] G.Guigas, M.Weiss, Biophys. J. 94, 90 (2008).

[B] S.M.J.Khadem et al., Phys. Rev. Research 3, 023169 (2021).

[C] E.Barkai, R.Silbey, J. Phys. Chem. B 104, 3866 (2000).

References

[1] Rey-Barroso J, Calovi DS, Combe M, German Y, Moreau M, Canivet A, et al. Switching between individual and collective motility in B lymphocytes

is controlled by cell-matrix adhesion and inter-cellular interactions. Sci Rep. 2018;8(1):5800. doi:10.1038/s41598-018-24222-4.

[2] Maiuri P, Rupprecht JF, Wieser S, Ruprecht V, Bénichou O, Carpi N, et al. Actin flows mediate a universal coupling between cell speed and cell persistence. Cell. 2015;161(2):374–386. doi:10.1016/j.cell.2015.01.056.

[3] Wortel I, Niculescu I, Kolijn M, Gov N, de Boer R, Textor J. Both cell-intrinsic and environmental factors constrain speed and persistence in T cell migration. bioRxiv. 2018; p. 338459. doi:10.1101/338459.

[4] Schiller HB, Hermann MR, Polleux J, Vignaud T, Zanivan S, Friedel CC, et al. β 1 - And α v -class integrins cooperate to regulate myosin II during rigidity sensing of fibronectin-based microenvironments. Nat Cell Biol. 2013;15(6):625–636. doi:10.1038/ncb2747.

[5] DeMali KA, Barlow CA, Burridge K. Recruitment of the Arp2/3 complex to vinculin: coupling membrane protrusion to matrix adhesion. J Cell Biol. 2002;159(5):881–91. doi:10.1083/jcb.200206043.

[6] Giannone G, Dubin-Thaler BJ, Rossier O, Cai Y, Chaga O, Jiang G, et al. Lamellipodial Actin Mechanically Links Myosin Activity with Adhesion-Site Formation. Cell. 2007;128(3):561–575. doi:10.1016/J.CELL.2006.12.039.

[7] Stanley P, Smith A, McDowall A, Nicol A, Zicha D, Hogg N. Intermediate-affinity LFA-1 binds α-actinin-1 to control migration at the leading edge of the T cell. EMBO J. 2008;27(1):62–75. doi:10.1038/sj.emboj.7601959.

[8] Copos CA, Walcott S, del Álamo JC, Bastounis E, Mogilner A, Guy RD. Mechanosensitive Adhesion Explains Stepping Motility in Amoeboid Cells. Biophys J. 2017;112(12):2672–2682. doi:10.1016/j.bpj.2017.04.033.
